# Dioleoylphosphatidylglycerol Inhibits Heat Shock Protein B4 (HSPB4)-Induced Inflammatory Pathways In Vitro

**DOI:** 10.3390/ijms24065839

**Published:** 2023-03-19

**Authors:** Teresa E. Fowler, Vivek Choudhary, Samuel Melnyk, Mishma Farsi, Luke Y. Chang, Nyemkuna Fortingo, Xunsheng Chen, Mitchell A. Watsky, Wendy B. Bollag

**Affiliations:** 1 Department of Ophthalmology, Medical College of Georgia at Augusta University, Augusta, GA 30912, USA; 2 Department of Physiology, Medical College of Georgia at Augusta University, Augusta, GA 30912, USA; 3 Charlie Norwood VA Medical Center, Augusta, GA 30904, USA; 4 Department of Cellular Biology and Anatomy, Medical College of Georgia at Augusta University, Augusta, GA 30912, USA; 5 James and Jean Culver Vision Discovery Institute, Medical College of Georgia at Augusta University, Augusta, GA 30912, USA

**Keywords:** diabetes, cluster of differentiation-14 (CD14), cornea, epithelium, heat shock protein B4 (HSPB4), high mobility group box 1 (HMGB1), inflammation, phosphatidylglycerol, toll-like receptor (TLR)

## Abstract

Our previous work shows that dioleoylphosphatidylglycerol (DOPG) accelerates corneal epithelial healing in vitro and in vivo by unknown mechanisms. Prior data demonstrate that DOPG inhibits toll-like receptor (TLR) activation and inflammation induced by microbial components (pathogen-associated molecular patterns, PAMPs) and by endogenous molecules upregulated in psoriatic skin, which act as danger-associated molecular patterns (DAMPs) to activate TLRs and promote inflammation. In the injured cornea, sterile inflammation can result from the release of the DAMP molecule, heat shock protein B4 (HSPB4), to contribute to delayed wound healing. Here, we show in vitro that DOPG inhibits TLR2 activation induced in response to HSPB4, as well as DAMPs that are elevated in diabetes, a disease that also slows corneal wound healing. Further, we show that the co-receptor, cluster of differentiation-14 (CD14), is necessary for PAMP/DAMP-induced activation of TLR2, as well as of TLR4. Finally, we simulated the high-glucose environment of diabetes to show that elevated glucose levels enhance TLR4 activation by a DAMP known to be upregulated in diabetes. Together, our results demonstrate the anti-inflammatory actions of DOPG and support further investigation into its development as a possible therapy for corneal injury, especially in diabetic patients at high risk of vision-threatening complications.

## 1. Introduction

When normal corneal tissue suffers an injury, damaged epithelial cells and stromal keratocytes release damage-associated molecular patterns (DAMPs), endogenous molecules that bind pattern recognition receptors such as toll-like receptors (TLRs) to activate the innate immune system. TLRs are also activated by microbial pathogen-associated molecular patterns (PAMPs), such as lipopolysaccharide (LPS), on the surface of gram-negative bacteria. The activation of TLRs triggers an intracellular cascade, culminating in the production of inflammatory cytokines and chemokines to propagate the immune response and recruit additional effectors [1,2]. Usually, this process occurs and resolves rapidly with the concomitant recovery of corneal clarity, while in some individuals, impaired healing and/or persistent inflammation can result in corneal ulceration or opacification. Heat shock protein B4 (HSPB4) is one DAMP shown to be released upon corneal injury and contribute to sterile inflammation, that is, inflammation in the absence of infection [3]. This can promote neutrophil infiltration into the cornea and decrease its transparency.

Voelker and colleagues have shown that in the lung, PAMP-induced TLR activation is inhibited by the phospholipid phosphatidylglycerol (PG) naturally present in surfactant [4]. We later demonstrated in cultured mouse macrophages and epidermal keratinocytes, as well as in a live mouse model of skin inflammation, that PG, and in particular dioleoyl-PG (DOPG), also inhibits the activation of TLRs induced by DAMPs active in psoriasis [5]. Further, we have recently shown that DOPG accelerates corneal wound healing both in vitro and in vivo [6], although this mechanism is still under investigation. Intriguingly, the DAMP-TLR interaction is aided by coreceptors, such as cluster of differentiation-14 (CD14) and myeloid differentiation protein-2 (MD2), which have also been shown to bind PG [7]. We hypothesized that DOPG inhibits activation of TLRs by DAMPs involved in corneal wound healing, like HSPB4, and that this inhibition improves corneal wound healing in inflammatory states.

One disease associated with delayed wound healing is diabetes, which is estimated to affect 10.5% of adults worldwide, or 536 million individuals, and is expected to rise to a prevalence of 12.2% in the next 25 years [8]. As the prevalence of diabetes increases, new therapies for its complications are needed. The complications of diabetes are recognized in every organ system, although ocular manifestations are among the most feared, as diabetic eye disease is among the most common causes of new blindness in adults [9]. The cornea is not immune to the destruction of diabetes, with up to 70% of diabetic patients affected by keratopathy [10,11,12]. Corneal injury in individuals with diabetes can be clinically challenging due to pain, delayed healing, neurotrophic keratitis, recurrent erosions, and superimposed infections. It is thought that changes in the inflammatory response play a significant role in corneal morbidity from diabetes.

High mobility group box 1 (HMGB1), a DAMP known to be elevated in diabetes [13], is released upon corneal injury [14] and also acts via TLR4 to initiate inflammation [15,16,17,18]. HMGB1, TLR2, TLR4, and several downstream ligands are upregulated in the corneas of diabetic animal models [19,20,21], and the inhibition of HMGB1 has been shown to improve diabetic mouse corneal healing [21]. To examine the effects on TLR activation of the corneal-relevant DAMPs, HSPB4 and HMGB1, as well as PAMPs as positive controls, we used a mouse macrophage cell line and a human epithelial kidney reporter cell line to demonstrate inhibition of TLR activation by DOPG, as well as by a neutralizing antibody to CD14. We also provide evidence that hyperglycemia enhances the activation of TLR4 by HMGB1, while having no effect on TLR2 activation by HSPB4; this effect may contribute to slow corneal recovery in diabetic patients. Our results support further exploration into DOPG as a therapeutic option for corneal injury due to its promotion of wound healing [6] and inhibition of inflammation (this study), with the goal of improving visual health and quality of life for diabetic patients.

## 2. Results

### 2.1. DOPG Inhibited Inflammatory Mediator Expression and Production in Mouse Macrophages Stimulated by HSPB4 and Human Corneal Epithelial Cells Exposed to a TLR2 Agonist

Studies in rodent models of sterile corneal inflammation upon injury have identified keratocyte-derived HSPB4 as an important DAMP that binds TLR2 on resident macrophages, initiating nuclear factor kappa-light-chain-enhancer of activated B cells (NF-κB) signaling to produce interleukins (ILs) and tumor necrosis factor-alpha (TNFα) [3]. In previous work, we have shown that DOPG inhibits inflammation in mouse skin in vivo [22], as well as in keratinocytes [23] and RAW264.7 mouse macrophage cells, by inhibiting TLR activation by the DAMP S100A9 [5]. In epidermal keratinocytes stimulated by recombinant S100A9, DOPG inhibits IL1β, IL6, and TNFα production [5]. We hypothesized that the upregulation of these factors in response to the activation of TLR2 by the DAMP, HSPB4, would also be inhibited by DOPG.

**Figure 1:**
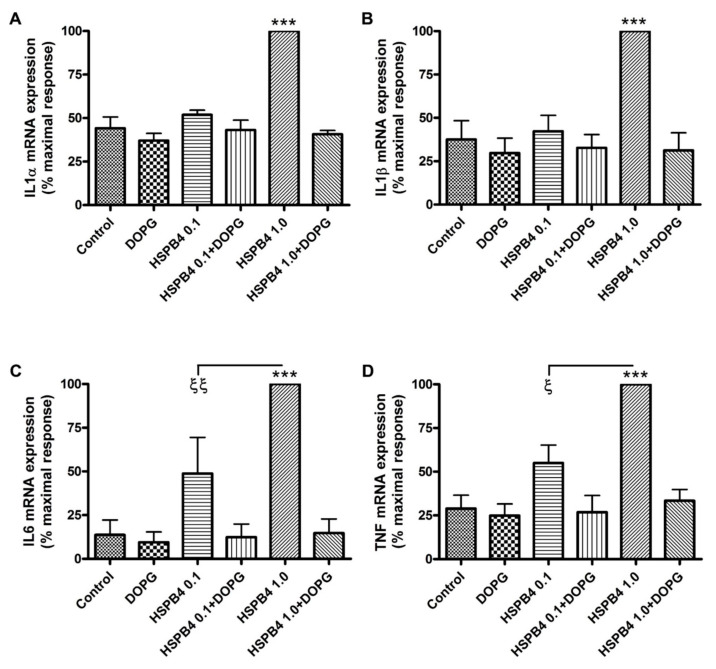
Upregulation of inflammatory mediator expression by recombinant HSPB4 is inhibited by DOPG in a macrophage cell line. RAW264.7 cells were treated with HSPB4 at 0, 0.1, or 1 µg/mL for 2 h in the presence or absence of 100 µg/mL DOPG. We performed qRT-PCR on RNA isolated from these cells to quantify expression levels of (**A**) IL1α, (**B**) IL1β, (**C**) IL6, and (**D**) TNF. GAPDH was selected for use as the housekeeping gene. Each experiment was performed in triplicate, with the results shown indicating the mean ± standard error of the mean for the three replicates. For each experiment, the highest expression level quantified by qRT-PCR, in each case observed in the presence of 1 µg/mL HSPB4, was set as “100% maximal”, and all others are shown as a percentage of that value. *** *p* < 0.001 versus all other values in the panel; ^ξ^ *p* < 0.05, ^ξξ^ *p* < 0.01 as indicated, using analysis of variance (ANOVA) with Tukey’s post-hoc testing. DOPG, dioleoylphosphatidylglycerol; HSPB4, heat shock protein B4; IL, interleukin; TNF, tumor necrosis factor.

**Figure 2:**
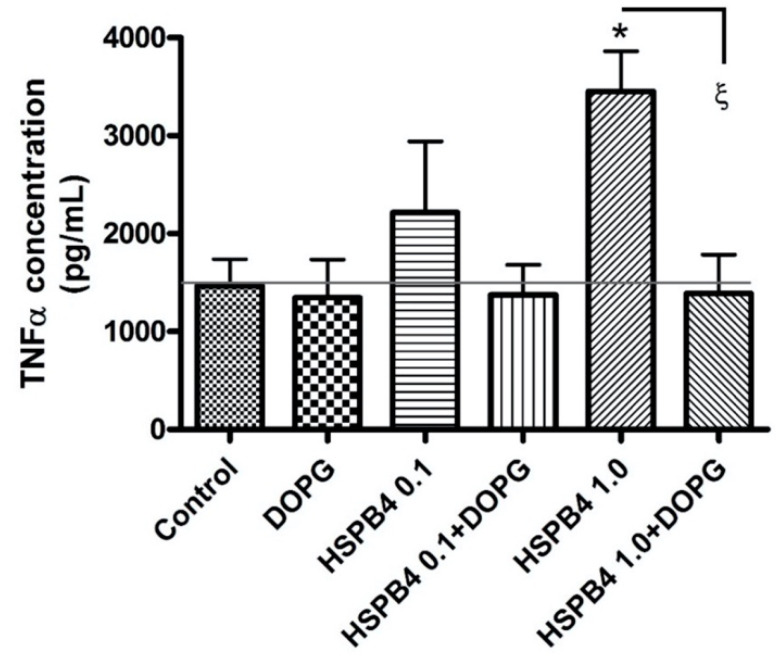
DOPG inhibited TNFα protein production in a macrophage cell line stimulated with recombinant HSPB4. RAW264.7 cells were treated with 0, 0.1, or 1 µg/mL HSPB4 in the presence or absence of 100 µg/mL DOPG for 2 h. ELISA was used to assess TNFα levels (pg/mL) in media collected from the treated cells. Experiments were performed on three separate occasions, with the results shown representing the means ± standard error of the mean. * *p* < 0.05 versus control; ^ξ^ *p* < 0.05 as indicated, using ANOVA with Tukey’s post-hoc testing. DOPG, dioleoylphosphatidylglycerol; HSPB4, heat shock protein B4; TNFα, tumor necrosis factor-α.

In Figure 1, transcriptional upregulation of inflammatory mediators was observed in response to HSPB4 in mouse macrophages, specifically of IL-1α, -1β, and -6, as well as TNF. In the presence of the lowest concentration of HSPB4 tested, 0.1 µg/mL, IL1α, and IL1β were not significantly upregulated, while IL6 and TNF were only moderately increased. With 1 µg/mL HSPB4, the transcription of all four mediators increased to a value that was statistically significantly different (*p* < 0.001) from all of the other values in the panels, with IL-1α increasing approximately two-fold, IL-1β rising about three-fold, IL-6, approximately five-fold and TNF, about four-fold. These results indicate that the response to HSPB4 is dose dependent. DOPG (at 100 µg/mL) counteracted this response, with the mRNA levels of all tested mediators returning to basal levels, i.e., values that were not significantly different from the control. In Figure 2, we demonstrate by ELISA that the observed increase in TNF mRNA was translated into elevated protein levels, as treatment with 1.0 µg/mL HSPB4 enhanced TNFα protein concentration by more than two-fold (*p* < 0.05). As expected, DOPG inhibited this response, again to a level that was not significantly different from the control value.

**Figure 3:**
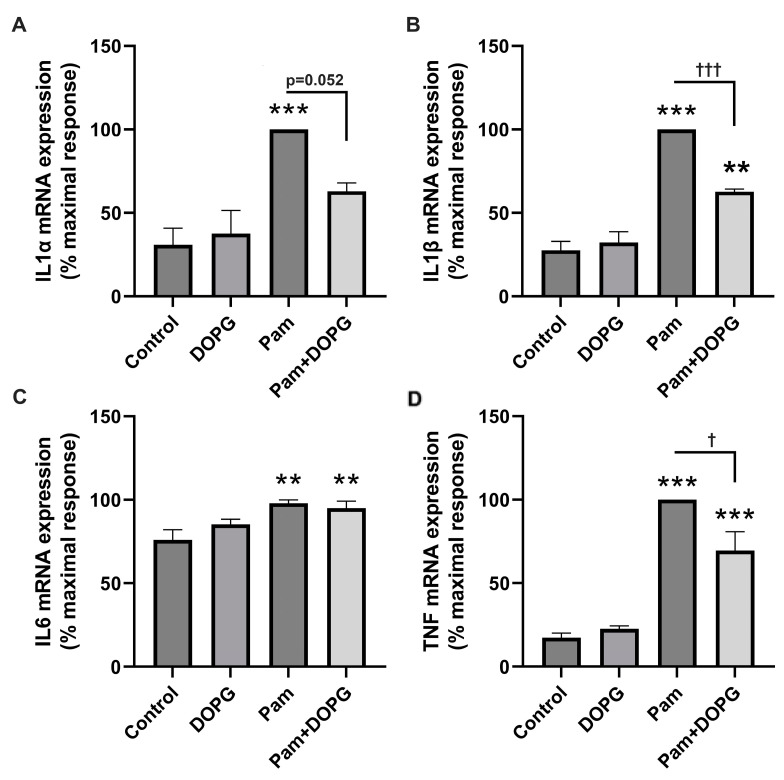
Upregulation of inflammatory mediator expression by a TLR2 agonist is inhibited by DOPG in normal human corneal epithelial cells (HCEC). HCEC were treated with or without 2.5 µg/mL Pam_3_(CSK)_4_ (Pam) for 2 h in the presence or absence of 100 µg/mL DOPG. We performed qRT-PCR on RNA isolated from these cells to quantify expression levels of (**A**) IL1α, (**B**) IL1β, (**C**) IL6, and (**D**) TNF. GAPDH and Rplp0 were selected as housekeeping genes (the mean C_t_ value of the two genes was used in delta–delta C_t_ calculations). Experiments were performed on four separate occasions, with results shown representing the means ± standard error of the mean. For each experiment, the highest expression level quantified by qRT-PCR, in each case observed in the presence of Pam, was set as “100% maximal”, and all others are shown as a percentage of that value. ** *p* < 0.01, *** *p* < 0.001 versus the control; ^†^ *p* < 0.05, ^†††^ *p* < 0.005 as indicated, using analysis of variance (ANOVA) with Tukey’s post-hoc testing. DOPG, dioleoylphosphatidylglycerol; HCEC, human corneal epithelial cells; IL, interleukin; Pam, Pam_3_(CSK)_4_; TNF, tumor necrosis factor.

We also determined the ability of DOPG to inhibit TLR2 activation in corneal epithelial cells. Normal human corneal epithelial cells (HCEC) between passages 3 and 7 were treated with the synthetic triacylated lipopeptide TLR2 agonist, Pam_3_(CSK)_4_ (Pam), and inflammatory mediator mRNA levels were assessed by quantitative RT-PCR. As shown in Figure 3, Pam increased the expression of three of the four inflammatory cytokines measured, by about three-fold (IL1α), four-fold (IL1β), and four-fold (TNF). As with TLR2 activated by HSPB4 in the macrophage cell line, DOPG inhibited this expression, returning mRNA levels of IL1α to values that were not significantly different from the control values and significantly inhibiting the expression of IL1β and TNF (by about 40% and 25%, respectively). The effect of Pam on IL6 expression, although significant, was minimal and was not altered by DOPG.

### 2.2. HSPB4 Dose-Dependently Induced TLR2 Activation in Human Cells, and DOPG Inhibited This Response

**Figure 4:**
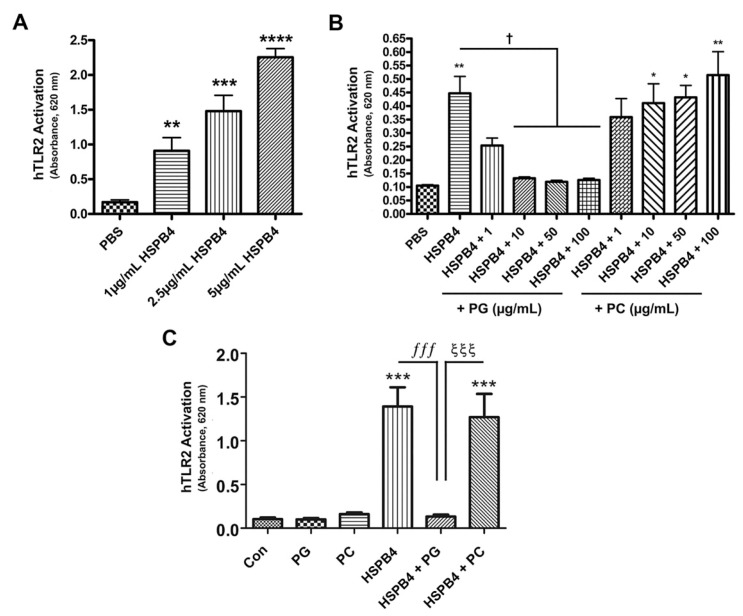
The HSPB4-induced activation of TLR2 was inhibited by DOPG, but not DOPC, at low concentrations. hTLR2 activity was quantified as the change in the absorbance of SEAP detection medium at 620 nm. Experiments were performed on three separate occasions, with results representing the means ± standard error of the mean. (**A**) hTLR2-expressing cells were treated with 1, 2.5, or 5 µg/mL recombinant HSPB4 for 24 h. HSPB4 activated TLR2 in a dose-dependent manner. ** *p* < 0.01, *** *p* < 0.001, **** *p* < 0.0001 versus the control (PBS). (**B**) Cells expressing human TLR2 receptors were treated with 1 µg/mL recombinant HSPB4 in the presence or absence of 1, 10, 50, or 100 µg/mL DOPG or DOPC. DOPG, but not DOPC, inhibited HSPB4-induced TLR2 activation at all tested concentrations. * *p* < 0.05, ** *p* < 0.01 versus PBS control; ^†^ *p* < 0.05 versus HSPB4 alone. (**C**) hTLR2 cells were treated with 10 µg/mL DOPG or DOPC in the presence or absence of 1 µg/mL recombinant HSPB4. DOPG, but not DOPC, inhibited hTLR2 activation by HSPB4. *** *p* < 0.001 versus the control (PBS); ^ξξξ^ *p* < 0.001; *^fff^ p* < 0.001 as indicated, using ANOVA with Tukey’s post-hoc testing. hTLR2, human toll-like receptor 2; HSPB4, heat shock protein B4; PBS, phosphate-buffered saline; PG, dioleoylphosphatidylglycerol; PC, dioleoylphosphatidylcholine.

To further investigate the mechanism behind the ani-inflammatory properties of DOPG, we next examined the activation of TLR2, the macrophage surface receptor for HSPB4. We used human reporter cells (HEK-Blue-hTLR2) which stably express TLR2, coreceptors CD14 and MD2, and a reporter construct which, when activated by TLR2 signaling, drives production of a secreted alkaline phosphatase (SEAP) detectable using the chromophore in the HEK-Blue Detection medium as a change in absorbance at 620 nm. In Figure 4A, we show dose-dependent activation of hTLR2 by recombinant HSPB4 using concentrations of 1, 2.5, and 5 µg/mL HSPB4, with the highest concentration of HSPB4 inducing an approximate 10-fold enhancement of TLR2 activation, as measured by the increase in absorbance at 620 nm (*p* < 0.0001).

Based on DOPG’s inhibition of TLR2 activation by HSPB4 in mouse macrophages, we anticipated a similar response in the human reporter cell line. Our previous experiments examined concentrations of 25, 50, and 100 µg/mL DOPG [6,24], with lower doses generally showing a similar efficacy as higher. Therefore, we elected to perform a dose response curve to observe the effect of DOPG on HSPB4’s activation of TLR2. Dioleoylphosphatidylcholine (DOPC), a phospholipid with the same two fatty acid tails as DOPG but a distinct head group, was used as a control based on previously published data that DOPC has little or no effect on these pathways [5]. HSPB4-activated hTLR2 cells were treated with DOPG at concentrations of 1, 10, 50, and 100 µg/mL. As shown in Figure 4B, 1 µg/mL HSPB4 induced an approximate 4.5-fold increase in TLR2 activation. In these HSPB4-exposed cells, the inhibitory effect of DOPG on TLR2 activation was robust even at the lowest tested concentration (1 µg/mL), with a return of hTLR2 activity to levels not significantly different from the control value, and maximal inhibition at approximately 10 µg/mL. Therefore, we repeated the experiment with this lower dose and included PG- and PC-alone groups, as shown in Figure 4C. We found that hTLR2 cells treated with 10 µg/mL DOPG or DOPC alone demonstrated hTLR2 activity equivalent to control cells. TLR2 activity was significantly upregulated (more than 10-fold, *p* < 0.001) in the presence of 1 µg/mL HSPB4, with DOPG, but not DOPC, blocking the stimulatory effect of HSPB4.

### 2.3. HMGB1 Activated TLR4, and DOPG, but Not DOPC, Inhibited This Activation

**Figure 5:**
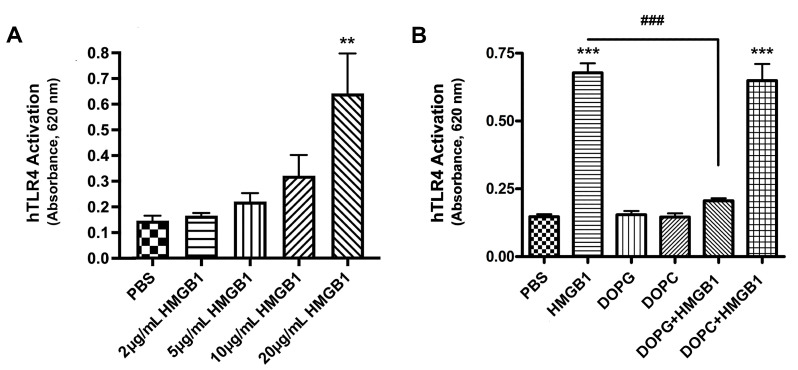
HMGB1-induced activation of TLR4 was inhibited by DOPG but not DOPC. SEAP was measured as absorbance at 620 nm to indicate hTLR4 activity. Experiments were performed on three separate occasions, with results representing the mean ± standard error of the mean of the absorbance. (**A**) HEK-Blue-hTLR4 cells were incubated for 24 h with phosphate buffered saline (PBS, control) or recombinant HMGB1 at the indicated concentrations. hTLR4 activation by HMGB1 was dose dependent. ** *p* < 0.01 versus control (PBS). (**B**) HEK-Blue-hTLR4 cells were incubated with or without 15 µg/mL HMGB1 in the presence or absence of 10 µg/mL DOPG or DOPC. DOPG, but not DOPC, inhibited the activation of hTLR4 by HMGB1. *** *p* < 0.001 versus control (PBS); ^###^ *p* < 0.001 as indicated, using ANOVA with Tukey’s post-hoc testing. hTLR4, human toll-like receptor 4; HMGB1, high mobility group box 1; PBS, phosphate-buffered saline; DOPG, dioleoylphosphatidylglycerol; DOPC, dioleoylphosphatidylcholine.

A growing body of evidence demonstrates that diabetes is a chronic inflammatory state. HMGB1 is an endogenous DAMP known to be upregulated in the serum of patients with diabetes [25]. Further, HMGB1 expression has been shown to be inhibited by metformin [26], a medication with anti-inflammatory properties [27] commonly used to manage diabetes. Additionally, HMGB1 has been shown to be upregulated after wounding, and its inhibition improves corneal wound healing [14]. We hypothesized that HMGB1 contributes to the impaired corneal wound healing seen in diabetic patients, and that DOPG would reduce this inflammatory stimulus. First, we investigated whether HMGB1 can activate TLR4 and if so, whether DOPG inhibited such activation. In Figure 5A, we show that using HEK-Blue-hTLR4 reporter cells, HMGB1 does indeed activate TLR4 in a dose-dependent manner. HMGB1 did not significantly activate TLR4 at 2 and 5 µg/mL, but it led to a modest response at 10 µg/mL, and significantly upregulated TLR4 activity at 20 µg/mL, with about a four-fold increase in hTLR4 activation at this highest dose (*p* < 0.01). In subsequent experiments we demonstrate that DOPG, but not DOPC, inhibited HMGB1-induced TLR4 activation, from a level that was approximately five-fold greater than the control (*p* < 0.001) to a value that was not significantly different from the control (Figure 5B).

### 2.4. HMGB1-Induced TLR4 Activation Was Increased in High Glucose Conditions but Ligand-Induced TLR2 Activation Was Not

**Figure 6:**
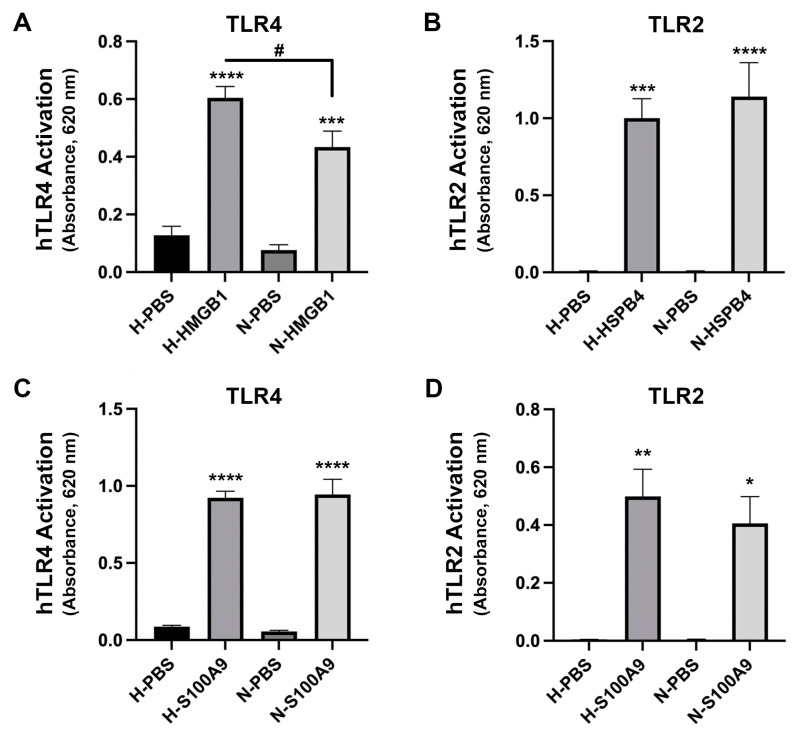
TLR4 activation by HMGB1, but not S100A9, increased in the presence of high glucose, but TLR2 activation by HSPB4 and S100A9 was unaffected. TLR activity was measured as a change in absorbance at 620 nm. Results represent the means ± standard error of the mean for four separate experiments. (**A**) HEK-Blue-hTLR4 cells were incubated with or without 20 µg/mL HMGB1 in growth medium containing either 1 g/L (Normal, N-) or 4.5 g/L (High, H-) glucose. Activation of TLR4 by HMGB1 was enhanced in high glucose. (**B**) HEK-Blue-hTLR2 cells were incubated with or without HSPB4 in growth medium containing either 1 g/L (Normal, N-) or 4.5 g/L (High, H-) glucose. hTLR2 activation was not significantly different between the two groups. (**C**,**D**) HEK-Blue-TLR4 (**C**) or HEK-Blue-TLR2 (**D**) cells were incubated with or without 10 µg/mL S100A9 in growth medium containing either 1 g/L (Normal, N-) or 4.5 g/L (High, H-) glucose. Activity of both TLR4 and TLR2 in response to S100A9 was equivalent in H- and N-glucose media. * *p* < 0.05, ** *p* < 0.01, *** *p* < 0.001, **** *p* < 0.0005 versus H- or N-PBS control; ^#^ *p* < 0.05 as indicated, using ANOVA with Tukey’s post-hoc testing. hTLR4, human toll like receptor 4; HMGB1, high mobility group box 1; HSPB4, heat shock protein B4; N-, normal glucose (1 g/L); H-, high glucose (4.5 g/L); hTLR2, human toll-like receptor 2; S100A9, S100 calcium binding protein A9.

To examine the effect of the hyperglycemia accompanying diabetes on the activation of TLR4 by HMGB1, we performed similar experiments in medium with high (4.5 g/L)—or normal (1 g/L)-glucose concentration. TLR4 activity was increased moderately in normal glucose (N-) medium but was significantly enhanced in the presence of high glucose (H-) (Figure 6A). In additional studies, we determined that this effect of the high-glucose medium was also observed in comparison with normal-glucose medium in which the osmolarity was matched to the high-glucose medium by the addition of mannitol (Supplemental Figure S1). We did not observe the same ability of high-glucose medium to augment TLR2 activation by HSPB4; the increase in TLR2 activity was equivalent in normal- and high-glucose states (Figure 6B). It was unclear whether the TLR involved or the activator was responsible for this differing response; therefore, we also examined the response to S100A9, a DAMP that activates both TLR2 and TLR4. We found that high-glucose medium did not enhance the response of either TLR4 (Figure 6C) or TLR2 (Figure 6D) by S100A9 in comparison to normal-glucose medium, suggesting that the effect might be related to the activator. Nevertheless, this finding suggests that a hyperglycemic environment can contribute to some, but not all, inflammatory cascades in the cornea.

### 2.5. CD14 Was Required for Maximal Activation of TLR2 and TLR4 by PAMPs and DAMPs

#### 2.5.1. Anti-CD14 Antibody and DOPG Inhibited hTLR4 Activation by LPS and S100A9

LPS, the major virulence factor responsible for septic shock from gram negative bacteria [28], is a PAMP that activates TLR4. Macrophages are known to express the surface pattern recognition receptor CD14, a known coreceptor for TLR4 [29]. Specific PAMP inflammatory stimuli, such as LPS in the outer membrane of gram-negative bacteria, bind both TLRs and CD14 [30] to produce a robust macrophage response [31]. CD14 has also been shown to bind PG [32] with this interaction likely important in PG’s ability to inhibit TLR activation in the lung [7]. We suspected that the neutralization of CD14 with an anti-CD14 antibody would mitigate the stimulatory effect of PAMPs and DAMPs on TLR4 receptors, and possibly interfere with the anti-inflammatory effect of DOPG. Therefore, we next wanted to explore the mechanisms regulating TLR4 activation by examining the interaction between DOPG and an antibody capable of neutralizing CD14 activity on LPS-induced TLR4 activation.

**Figure 7:**
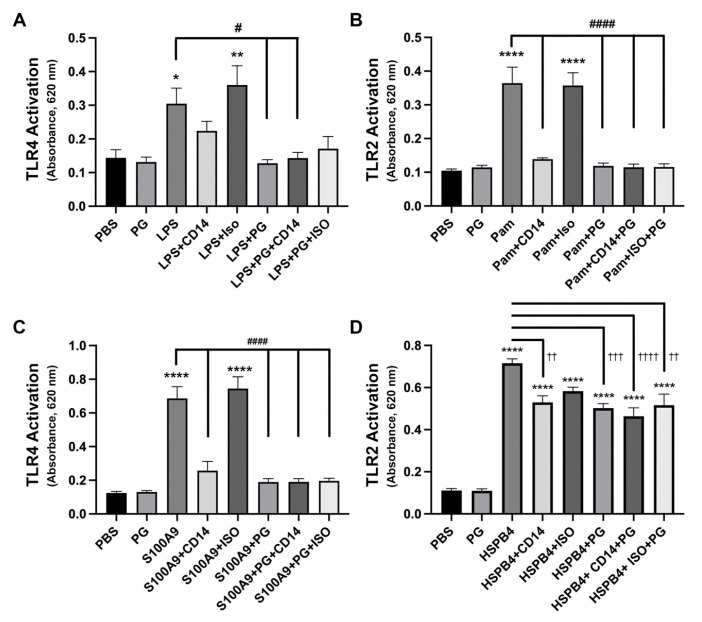
TLR4 activation by LPS and S100A9, and TLR2 activation by Pam and HSPB4, were inhibited by DOPG. This inhibition was not affected by the presence of a CD14-blocking antibody. The activity of secreted alkaline phosphatase as an indicator of TLR activity was measured as absorbance at 620 nm. Experiments were performed on three separate occasions, with results representing the means ± standard error of the mean. (**A**) HEK-Blue-hTLR4 reporter cells were incubated with 0.001 µg/mL LPS in the presence or absence of 100 µg/mL DOPG and/or 2.5 µg/mL anti-CD14 or Isotype control antibody (ISO). (**B**) HEK-Blue-hTLR2 reporter cells were incubated with 0.00003 µg/mL Pam in the presence or absence of 10 µg/mL DOPG and/or 5 µg/mL anti-CD14 or ISO. (**C**) HEK-Blue-hTLR4 cells were incubated with 3 µg/mL recombinant S100A9 in the presence or absence of 100 µg/mL DOPG and/or 2.5 µg/mL anti-CD14 antibody (CD14) or ISO. (**D**) HEK-Blue-hTLR2 reporter cells were incubated with 1 µg/mL HSPB4 in the presence or absence of 1 µg/mL DOPG and/or 1 µg/mL anti-CD14 or ISO. * *p* < 0.05, ** *p* < 0.01, **** *p* < 0.0005 versus the PBS control; ^#^ *p* < 0.05, ^####^ *p* < 0.0005, ^††^ *p* < 0.01, ^†††^ *p* < 0.001, ^††††^ *p* < 0.0005 as indicated, using ANOVA with Tukey’s post-hoc testing. hTLR4, human toll-like receptor-4; LPS, lipopolysaccharide; PBS, phosphate-buffered saline; CD14, antibody to cluster of differentiation-14; PG, dioleoylphosphatidylglycerol; ISO, isotype antibody; hTLR2, human toll-like receptor-2; Pam, Pam_3_Cys-Ser-(Lys)_4_; HSPB4, heat shock protein B4.

As shown in Figure 7, LPS significantly increased the activation of TLR4 in the reporter cell line approximately two-fold (*p* < 0.05), but 100 µg/mL DOPG blunted this response, with a return of TLR4 activity to control levels. When hTLR4 cells were treated with LPS in the presence of the anti-CD14 neutralizing antibody, TLR4 activation was again inhibited to a value not significantly different from the control, supporting the importance of CD14 in this interaction. Note that an antibody of the same isotype (ISO) as the anti-CD14 antibody (IgG1), but which does not recognize CD14, was used as a control for potential non-specific antibody effects, and as expected, did not inhibit LPS-induced TLR4 activation. Cells treated with both DOPG and anti-CD14 demonstrated low TLR4 activity that was not significantly different from either compound alone, making it difficult to evaluate whether an additive effect was present; however, there was also no interference with either’s inhibitory effect. Taken together, these data support an important role for the CD14 co-receptor in PAMP-induced activation of TLR4, as well as the ability of both DOPG and an anti-CD14 neutralizing antibody to inhibit this process.

S100 calcium-binding protein A9 (S100A9) is a DAMP released upon corneal wounding, which contributes to corneal inflammation [33]. S100A9 activates both TLR2 and TLR4 [34,35], and in RAW264.7 macrophages we have previously shown that DOPG inhibits S100A9-induced hTLR2 activation in the reporter cell line [5]. We next questioned whether TLR4 activation induced by DAMPs also requires CD14. In Figure 7B, we observed increased TLR4 activity in the presence of S100A9 (of about six-fold, *p* < 0.0001), with inhibition in the presence of the anti-CD14 antibody or DOPG to levels that were not significantly different from the control value. TLR4 reporter cells exposed to S100A9, anti-CD14, and DOPG concurrently demonstrated receptor activity equivalent to that of S100A9 with DOPG alone, again suggesting no interference from anti-CD14 towards the inhibitory effect of DOPG.

#### 2.5.2. TLR2 Activation by a PAMP or DAMP Was Inhibited by DOPG and by an Antibody Recognizing CD14, but Effects Were Not Additive

Although CD14 is well known to serve as a coreceptor for TLR4 [29], perhaps less recognized is its involvement in TLR2 activation. We first verified the importance of CD14 in the TLR2 response to PAMPs. We exposed hTLR2 cells to a synthetic microbial triacylated peptide known to activate TLR2, Pam_3_CSK_4_ (Pam) and, as expected, saw a significant rise in TLR2 activity to more than three-fold over the control (*p* < 0.0001). Pam stimulation of TLR2 activity was blunted by treatment with either anti-CD14 or DOPG alone, as well as in combination, to levels that were not significantly different from the control value (Figure 7C); again, the combination showed neither an additive nor a subtractive response.

We next wanted to know whether the same responses would occur when cells were stimulated with an endogenous DAMP, such as HSPB4, instead of a microbial PAMP. We also used a lower concentration of antibody and DOPG (both 1 µg/mL) to see if submaximal doses of these two agents would show additive effects. As shown in Figure 7D, we found that hTLR2 activity stimulated by HSPB4, which was elevated by approximately seven-fold (*p* < 0.0001), was inhibited by both DOPG and anti-CD14 antibody by about 33% but that there was no additive inhibitory effect of the combination. In another set of experiments, we used comparable doses as in Figure 7A–C (10 µg/mL DOPG and 2.5 µg/mL anti-CD14 antibody) and observed that hTLR2 activity stimulated by HSPB4 is equivalently inhibited by DOPG and anti-CD14 antibody, such that exposure to both inhibitors in combination yielded TLR2 activity not significantly different from either inhibitor alone.

### 2.6. DMPG, a Phosphatidylglycerol Already Used in Commercial Eye Drops, Has a Similar Inhibitory Effect as DOPG on HSPB4-Induced TLR2 Activation

**Figure 8:**
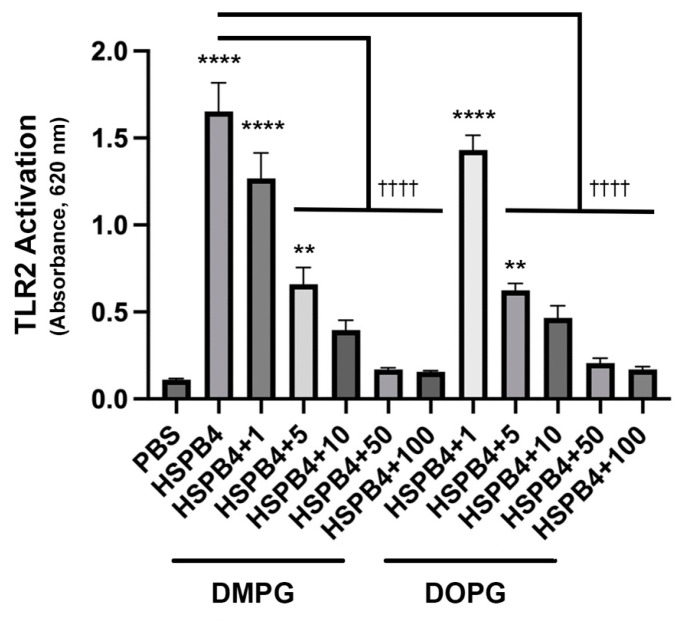
DMPG and DOPG both inhibited HSPB4-induced TLR2 activation. HEK-Blue-hTLR2 reporter cells were incubated with 1 µg/mL HSPB4 in the presence or absence of various concentrations of DMPG or DOPG as indicated (in µg/mL). In the presence of HSPB4, there was no significant difference in inhibition of HSPB4-mediated TLR2 activation between corresponding doses of DMPG and DOPG, and DMPG alone did not affect TLR2 activation. The activity of secreted alkaline phosphatase was measured as absorbance at 620 nm. Experiments were performed on three separate occasions, with results representing the means ± standard error of the mean. ** *p* < 0.01, **** *p* < 0.0005 versus the PBS control; ^††††^ *p* < 0.0005 as indicated, using ANOVA with Tukey’s post-hoc testing. hTLR2, human toll-like receptor-2; PBS, phosphate-buffered saline; DMPG, dimyristoyl-phosphatidylglycerol; DOPG, dioleoylphosphatidylglycerol; HSPB4, heat shock protein B4.

Alcon’s treatment for dry eye, Systane^®^ Complete Lubricant Eye Drops, lists dimyristoylphosphatidylglycerol (DMPG) as an inert ingredient [36]. DMPG is a species of phosphatidylglycerol with two saturated 14-carbon fatty acids in place of the two monounsaturated 18-carbon fatty acids found in DOPG. Importantly, Voelker and colleagues have previously shown that DMPG can block LPS-induced TLR4 activation in alveolar macrophages [7]. Therefore, DMPG was tested in the reporter cell system for its ability to inhibit TLR2 activation by HSPB4, since this DAMP is known to be released in the cornea upon wounding [3]. Figure 8 demonstrates that HSPB4 increased TLR2 activation by more than 10-fold (*p* < 0.0001) and DMPG inhibited HSPB4-induced TLR2 activation with a similar dose dependence as DOPG, such that doses of 5 µg/mL or greater DMPG/DOPG significantly reduced—and doses of 50 µg/mL and above completely blocked—TLR2 activation, returning the levels to a value not significantly different from the control. DMPG alone had no effect on TLR2 activation. Thus, although the concentration of DMPG in Systane^®^ Complete is not provided by the company, this result suggests the possibility that the DMPG in these eye drops may not be an inert ingredient, as previously assumed.

## 3. Discussion

Corneal injury involves complex inflammatory signaling, with activation of TLRs leading to production of cytokines and chemokines to recruit immune effector cells. In this work, we used a macrophage cell model and the HEK reporter cell line to investigate several inflammatory stimuli, including representative endogenous DAMPs and microbial PAMPs, along with their TLR receptors and CD14 coreceptor. We selected the RAW264.7 mouse macrophage cell line to model the stromal macrophages previously shown to respond to HSPB4 [3] and also examined the response of HCECs to a synthetic triacylated lipopeptide TLR2 agonist. We chose to use the HEK-Blue-hTLR2 and -hTLR4 reporter cells as highly sensitive indicators of TLR2 or TLR4 activation. For DAMPs, we selected HSPB4 based on previous studies demonstrating its importance in mediating the corneal keratocyte inflammatory response to epithelial damage [3]. Our interest in HMGB1 stemmed from its established upregulation in diabetes [25]. We show new evidence that CD14 is an important coreceptor in the activation of both TLR2 and TLR4, and that neutralization of CD14 dampens TLR-induced NF-κB activation, and presumably the immune response, as does treatment with DOPG. DOPG is a potent inhibitor of these pathways, even at low concentrations, as is DMPG, which we propose makes these phospholipids a promising therapeutic option for noninfectious corneal inflammation, especially in cases of impaired healing such as in diabetes.

In this study, we used high-glucose media to simulate the hyperglycemic state present in diabetes, finding enhancement of TLR4 activation by HMGB1 by the high glucose concentration but no effect on HSPB4-induced TLR2 activation. These results suggest that augmentation of specific inflammatory signaling interactions is one mechanism by which diabetes may contribute to prolonged inflammation, providing a potential target for development of therapeutic interventions. It should be noted that since the serum levels of HMGB1 are increased in diabetic individuals [13], TLR4 activation by this DAMP might also play a role in impaired skin wound healing in diabetes. Therefore, DOPG could potentially be applied topically to these wounds to reduce inflammation and promote wound healing.

Several properties of DOPG make it an attractive potential therapeutic. Previous work by our laboratory supports its efficacy in promoting healing of injured corneal epithelium both in vitro and in vivo [6]. PG is found naturally in human surfactant, and thus is presumably safe for use as a topical medication for humans. As mentioned in Section 2, PG, in the form of DMPG, which also inhibits HSPB4-induced TLR2 activation, is already present in an ophthalmic solution used to treat dry eye, indicating a good safety profile in this organ and stability in commercially available topical formulations. Further, PG suppresses DAMP- and PAMP-stimulated inflammation without suppressing immune protection globally, demonstrated by studies in the lung showing that inhaled PG enhances protection against viral infection [4,37,38], and thus, may be safe for use, even if an unrecognized infectious pathogens are present. The development of safe and effective drops with a favorable side effect profile to enhance recovery from and reduce vision-threatening complications of corneal disease could considerably improve visual outcomes, especially in diabetic patients.

The mechanism by which DOPG (or DMPG) functions to inhibit TLR activation is not entirely clear. In 2009, Voelker and colleagues showed that two components of the TLR signaling system, CD14, an apparent co-receptor, and myeloid differentiation factor-2 (MD-2), an adaptor protein, are able to bind phosphatidylglycerol [7], suggesting the possibility that one (or both) of these proteins mediates the effects of DOPG. A more recent study with phosphatidylglycerol analogs indicates that the capacity of these analogs to inhibit PAMP-induced TLR activation correlates with their binding to CD14, rather than MD-2 [32], identifying the CD14 TLR co-receptor as the likely site of DOPG’s action. Nevertheless, in the current report, the blocking antibody recognizing CD14 did not act additively, nor did it inhibit, the ability of DOPG to reduce TLR2 or TLR4 activation by either PAMPs or DAMPs. This result raises the question as to whether DOPG is actually working through CD14. However, the data do not eliminate the possibility that the anti-CD14 and DOPG bind to different sites on CD14 and the binding of the antibody and/or phospholipid does not change CD14’s conformation sufficiently to alter its capacity to bind the other.

At present, oral doxycycline is one of the limited therapeutic options employed in patients with prolonged corneal inflammation, including diabetic corneal injury. This broad-spectrum antibiotic has been shown to have anti-inflammatory properties in the cornea distinct from its antibiotic effects, including the inhibition of matrix metalloproteinases [39], blockage of immune cell activation, and reduction of unwanted neovascularization [40]. Similar to the effects seen with DOPG, topical doxycycline in mouse models of dry eye reduces expression of IL-1α, IL-1β, and TNF-α, and in LPS-stimulated corneal epithelial cells, it has been shown to reduce IL-1β transcription and translation [41]. Topical doxycycline has also recently been shown to inhibit TNFα expression in rat corneas subjected to alkali burn [42], and to block production of nitric oxide by cultured macrophages stimulated by LPS [43]. Of course, the use of systemic doxycycline can have unwanted effects unrelated to the eyes, such as gastrointestinal distress, esophagitis, photosensitivity, and hepatotoxicity, as well as the promotion of antibiotic resistance, and patients with corneal disease are often kept on this medication for long periods of time. Our results suggest that DOPG and DMPG have similar anti-inflammatory properties to doxycycline but without some of the unwanted side effects, supporting further investigation into these agents’ potential development as a therapeutic option.

Limitations of this study include the fact that the results were obtained in vitro, although this approach allowed us to investigate human TLR signaling with a highly sensitive reporter system, and to begin to examine the mechanisms by which DOPG might be exerting its anti-inflammatory effects. Nevertheless, it is unclear whether these same effects will occur in vivo. In addition, for our studies examining the ability of HSPB4 to stimulate inflammatory mediator production in innate immune cells, we used a macrophage cell line, RAW264.7 cells, which may not be a good equivalent of normal macrophages [44]. Finally, incubating cells in a high-glucose medium for a short time cannot reproduce the many changes experienced by tissues in a chronic disease like diabetes.

In summary, these results provide new insight into the anti-inflammatory effects of DOPG (and DMPG) in response to DAMPs and PAMPs. Our representative DAMPs were selected based on their role in corneal injury or in diabetes, which is known to be a chronic inflammatory state with impaired corneal healing [45,46,47]. We provide new evidence that neutralization of the CD14 coreceptor blunts TLR2 and TLR4 activation in response to DAMPs and PAMPs, but does not interfere with DOPG’s action. Next steps include in vivo application of this knowledge to mouse models of corneal injury, including knockouts with impaired healing and those with diabetes. The primary goal of our work is to identify potential novel therapeutics to reduce the morbidity of inflammatory corneal disease, and these results support DOPG as a promising candidate for development into a topical therapy.

## 4. Materials and Methods

### 4.1. Materials

Cultured RAW264.7 mouse macrophage cells were obtained from Dr. Carlos Isales (Augusta University, Augusta, GA, USA) who purchased them from American Type Culture Collection (Manassas, VA, USA). HEK-Blue-hTLR2 (catalog #hkb-htlr2) and -hTLR4 (catalog #hkb-htlr4) cells expressing CD14, MD2, and an inducible secreted embryonic alkaline phosphatase (SEAP) reporter gene were purchased from InvivoGen (San Diego, CA, USA) as was the HEK-Blue Detection medium (catalog #hb-det3). Enzyme linked immunosorbent assay (ELISA) studies were performed with kits obtained from BD Biosciences (San Jose, CA, USA). Recombinant human S100A9 protein (catalog #9254-S9-050) was from R&D Systems (Minneapolis, MN, USA) and Recombinant Human AlphaA Crystallin/CRYAA (HSPB4; NBC1-18351) from Novus Biologicals (Centennial, CO, USA). Phospholipids (DOPG, DOPC, and DMPG) were obtained from Avanti Polar Lipids, Inc. (Alabaster, AL, USA). Pam_3_Cys-Ser-(Lys)_4_ (Pam; catalog #506350) and lipopolysaccharide (LPS) were obtained from Sigma-Aldrich (St. Louis, MO, USA). Human CD14 antibody (MAB3832) and mouse IgG1 Isotype control monoclonal antibody (MAB002) were purchased from R&D Systems (Minneapolis, MN, USA).

### 4.2. Cell Culture

Culture medium for RAW264.7 cells consisted of Dulbecco’s Modified Eagle Medium (DMEM) with 10% fetal bovine serum and 1% penicillin and streptomycin. HEK-Blue-hTLR2 and hTLR4 cells were cultured in a growth medium, as per the supplier’s instructions. For experiments examining cells treated under normal-(1 g/L glucose) and high (4.5 g/L glucose)-glucose conditions, media were from Fisher Scientific (Hampton, NH, USA; catalog numbers MT10014CV and MT10013CM, respectively).

Primary human corneal epithelial cells (HCEC) were prepared from human corneal rims, as described in [48]. HCEC (passage 3–7) were maintained in normal-glucose (1 g/L) DMEM containing 10% fetal bovine serum, insulin/transferrin/selenium (ITS), epidermal growth factor (EGF) and gentamycin. For experiments, the HCEC were grown on 6-well plates with an initial plating density of 150,000 cells per well. Once the cells reached approximately 70% confluence, the cells were treated with or without the TLR2 agonist Pam (2.5 µg/mL) in the presence and absence of DOPG (100 µg/mL) for 2 h. At the end of the treatment, cells were collected in RNA lysis buffer (PureLink RNA Mini kit, Thermo Fisher Scientific) and total RNA was isolated and analyzed as described below.

### 4.3. Quantitative Real-Time Polymerase Chain Reaction (qRT-PCR)

After the treatment of cells, PerfectPure RNA tissue kits (QuantaBio, Gaithersburg, MD, USA) were used for total RNA extraction, followed by verification of RNA quality and quantity using a Nanodrop spectrophotometer (Wilmington, DE, USA). Reverse transcription of the total RNA was performed using iScript cDNA synthesis kits from Bio-Rad Laboratories (Hercules, CA, USA). Real time quantitative polymerase chain reaction (qRT-PCR) experiments were performed using Taqman probes (Supplemental Table S1), Fast Reagent PCR Master Mix, and the StepOnePlus Real-time PCR system from Applied Biosystems (Waltham, MA, USA). GAPDH was selected for use as the housekeeping gene and gene expression values calculated using the ΔΔC_t_ method. For the studies in HCEC, mRNA expression of inflammatory cytokines was analyzed using GAPDH and RPLP0 as reference genes. The data were expressed as the percent of the maximal response for each cytokine. 

### 4.4. Absorbance Assays to Assess TLR2 or 4 Activity

TLR2 and TLR4 activation was determined using human reporter cells (HEK-Blue-hTLR2 and HEK-Blue-hTLR4), which stably express human TLR2 or TLR4, the coreceptors CD14 and MD2, and a reporter construct which drives the production of a secreted alkaline phosphatase (SEAP) when activated by TLR signaling. SEAP activity can then be detected using HEK-Blue Detection medium, which contains a chromogenic substrate that changes its absorbance at 620 nm when acted upon by SEAP. Cultured HEK-Blue-hTLR2 or -hTLR4 reporter cells were resuspended in HEK-Blue Detection medium and added to a 96-well plate with the desired activator (HMGB1, HSPB4, LPS, Pam, or S100A9) and/or inhibitor (DOPG, anti-CD14 antibody). Phosphate-buffered saline (PBS), DOPC, and an isotype control antibody (ISO) were used as controls. Cells were treated for 24 h with the selected compounds at 37 °C. Absorbance was measured at 620 nm to determine secreted embryonic alkaline phosphatase (SEAP) activity, using a Synergy HT microplate reader from Bio-Tek Instruments (Winooski, VT, USA) with Gen5 analysis software.

### 4.5. ELISA Assay

Supernatants of treated RAW264.7 cells were collected and analyzed by an enzyme-linked immunosorbent assay (ELISA) from BD Biosciences (San Jose, CA, USA), as previously described [49].

### 4.6. Statistical Analysis

All experiments were repeated in at least duplicate in at least 3 separate experiments, with the results reported as the mean ± standard error of the mean. Differences between two groups were compared using an unpaired, two-tailed *t*-test. When three or more groups were analyzed, a one-way analysis of variance was used with a Tukey’s post-hoc test.

## 5. Conclusions

Corneal injuries in patients with diabetes can be slow to heal, and they are at higher risk for complications than in those without this disease. Since inflammation is thought to be one of the mechanisms by which diabetes impairs corneal wound healing, here, we investigated in vitro possible inflammatory mechanisms likely to be active in damaged corneal tissues using specific proteins known to be elevated in diabetes or upon corneal wounding. Further, we propose a naturally occurring phospholipid as a promising therapeutic candidate to promote corneal wound healing and reduce vision impairment resulting from corneal inflammation.

## Data Availability

All data are contained within this report.

## Supplementary Materials

The supporting information can be downloaded at: https://www.mdpi.com/article/10.3390/ijms24065839/s1.
