# Supplementary material for: Dioleoylphosphatidylglycerol Inhibits Heat Shock Protein B4 (HSPB4)-Induced Inflammatory Pathways In Vitro
Source: Int J Mol Sci. 2023 Mar 19;24(6):5839. doi: 10.3390/ijms24065839 (PMC10059050; doi:10.3390/ijms24065839)
Supplement: Supplementary file 1 [file ijms-24-05839-s001.zip › ijms-2078339-supplementary.pdf]

Supplemental Methods

<u>Controlling for Media Osmolarity</u>

To match the osmolarity between the normal-glucose and high-glucose medium, a high mannitol solution was used. The mannitol solution was prepared using D-mannitol at a molarity of 1.09786 (1097.86 mmol) (Sigma-Aldrich, St. Louis, MO) by dissolving the sugar alcohol in distilled deionized water and filter-sterilizing with a sterile 0.2µm pore syringe filter (Corning, Somerville, MA). Media osmolarity was measured using a VAPRO vapor pressure osmometer model 5600 (Wescor, Logan, Utah) according to the company's instructions. Initial osmolarity for both the normal- and high-glucose media was measured and appropriate amounts of the mannitol solution added to yield the same osmolarity in both media.

Table S1. Taqman® Gene Expression Assay Primer-Probe Sets for Quantitative RT-PCR

**Assay ID**	**Gene Symbol**	**Gene Name**	**Species**	**Context Sequence**
Mm00446190_m1	Il6	Interleukin-6	Mus musculus	TGAGAAAAGAGTTGTGCAATGGCAA
Mm00439620_m1	Il1a	Interleukin-1α	Mus musculus	ACCTGCAACAGGAAGTAAAATTTGA
Mm00434228_m1	Il1b	Interleukin-1β	Mus musculus	GACCCCAAAAGATGAAGGGCTGCTT
Mm00443258_m1	Tnf	Tumor necrosis factor	Mus musculus	CCCAAAGGGATGAGAAGTTCCCAAA
Mm99999915_g1	Gapdh	Glyceraldehyde 3-phosphate dehydrogenase	Mus musculus	GGTGTGAACGGATTTGGCCGTATTG

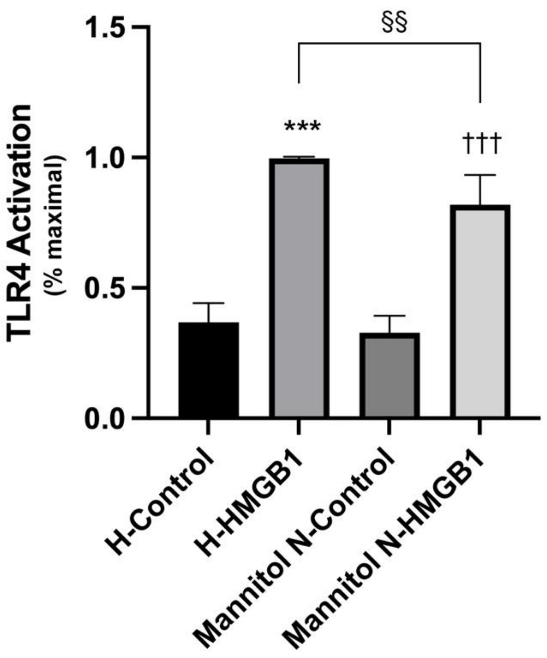

Supplemental Figure S1. High glucose medium enhanced TLR4 activation in response to HMGB1 compared to normal glucose medium matched for osmolarity. HEK-Blue-hTLR4 cells were incubated with or without 10 µg/mL HMGB1 in growth medium containing either high 4.5 g/L (H-) glucose or normal 1 g/L glucose with osmolarity matched to the high-glucose medium using mannitol (Mannitol N-). TLR4 activity was measured as a change in absorbance at 620 nm. Results expressed as the percent maximal response in each experiment represent the means ± standard error of the mean for 5 separate experiments. ***P<0.001 versus the high-glucose control; †††P<0.001 versus the mannitol-matched normal-glucose control; §§P<0.01 as indicated, using ANOVA with Tukey's multiple comparison post-hoc testing. TLR4, human toll like receptor 4; HMGB1, high mobility group box 1; Mannitol N-, normal-glucose (1 g/L) with mannitol added to match the osmolarity of the high-glucose medium; H-, high glucose (4.5 g/L).
